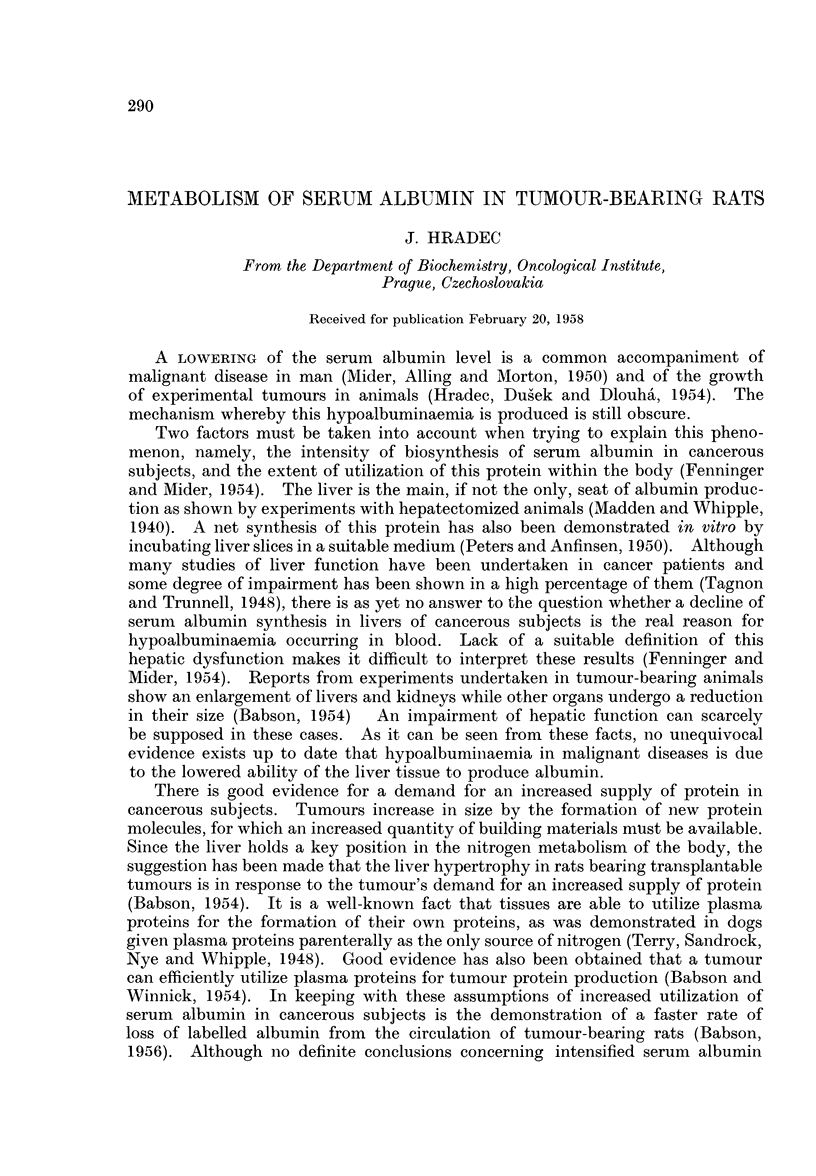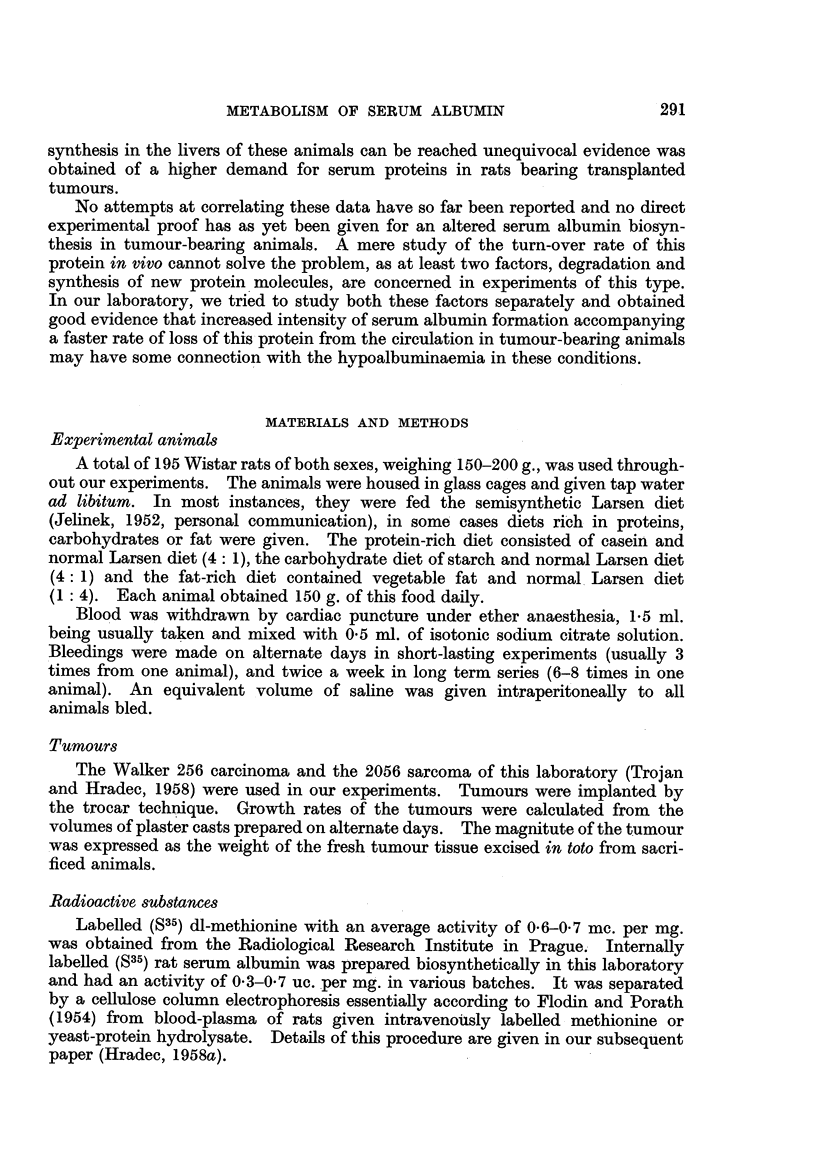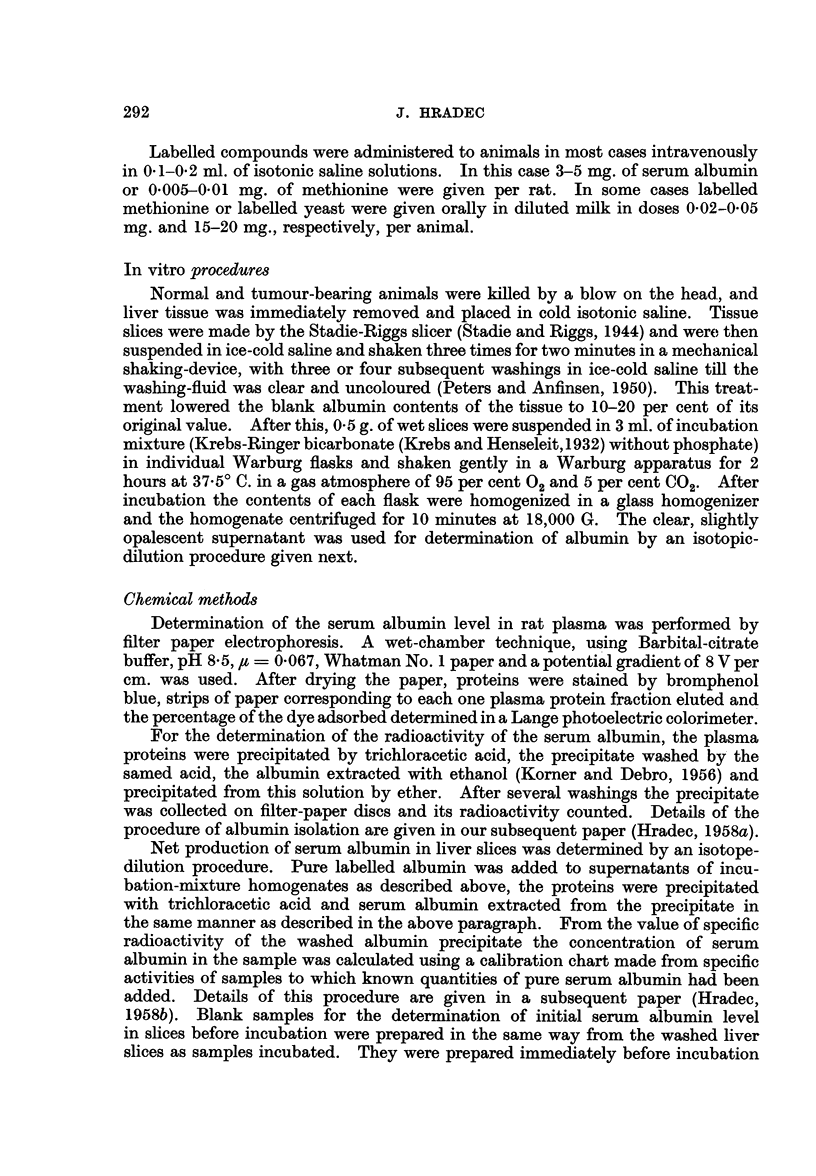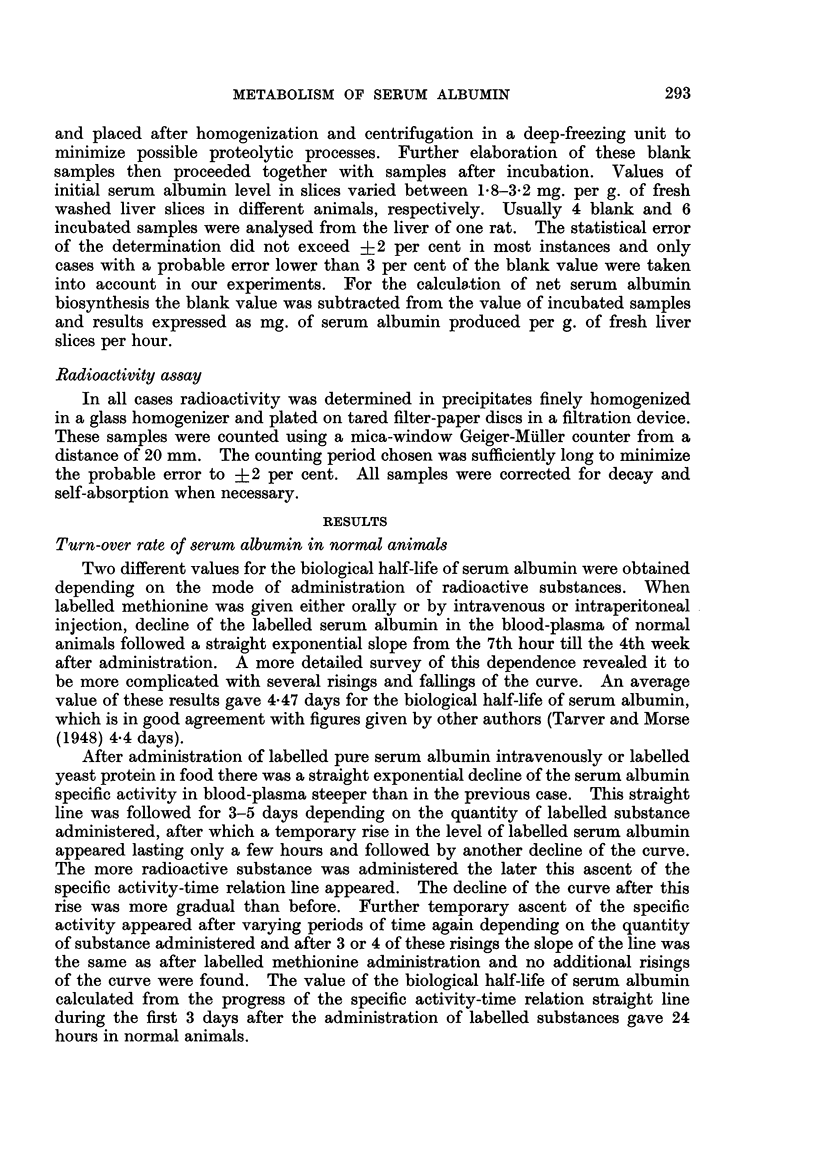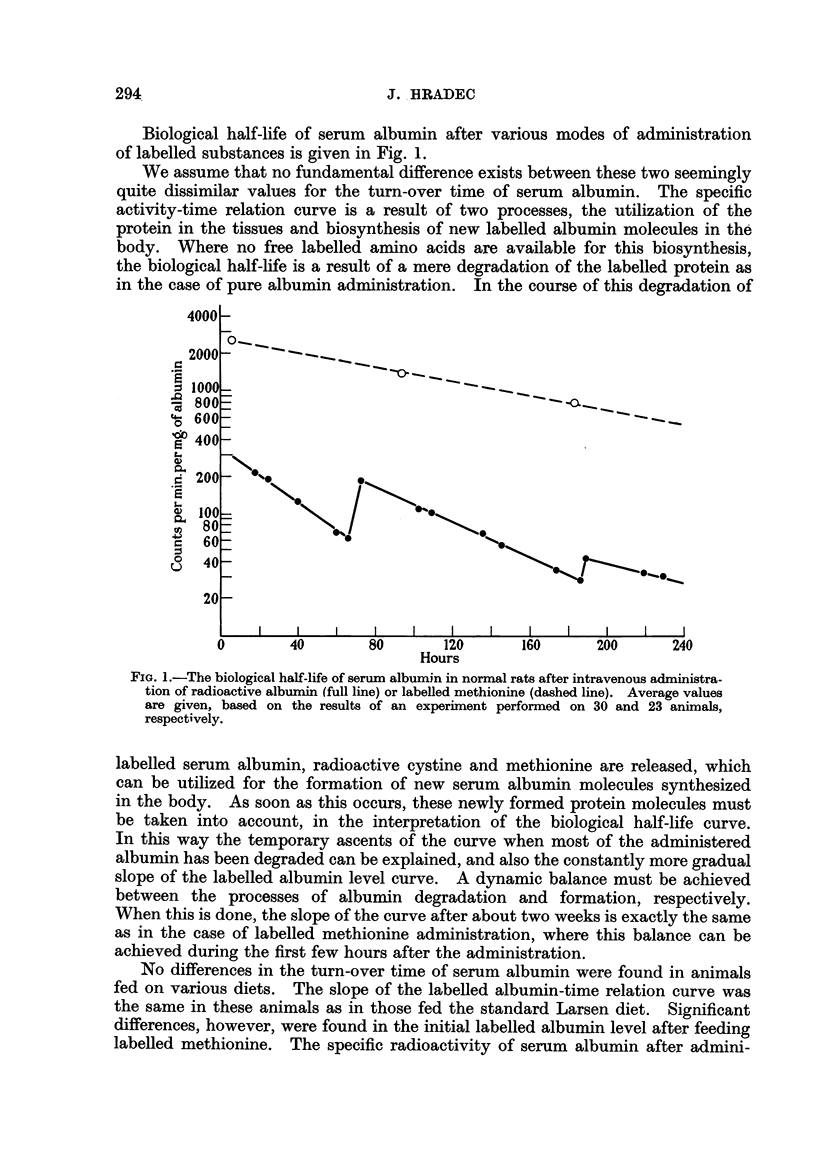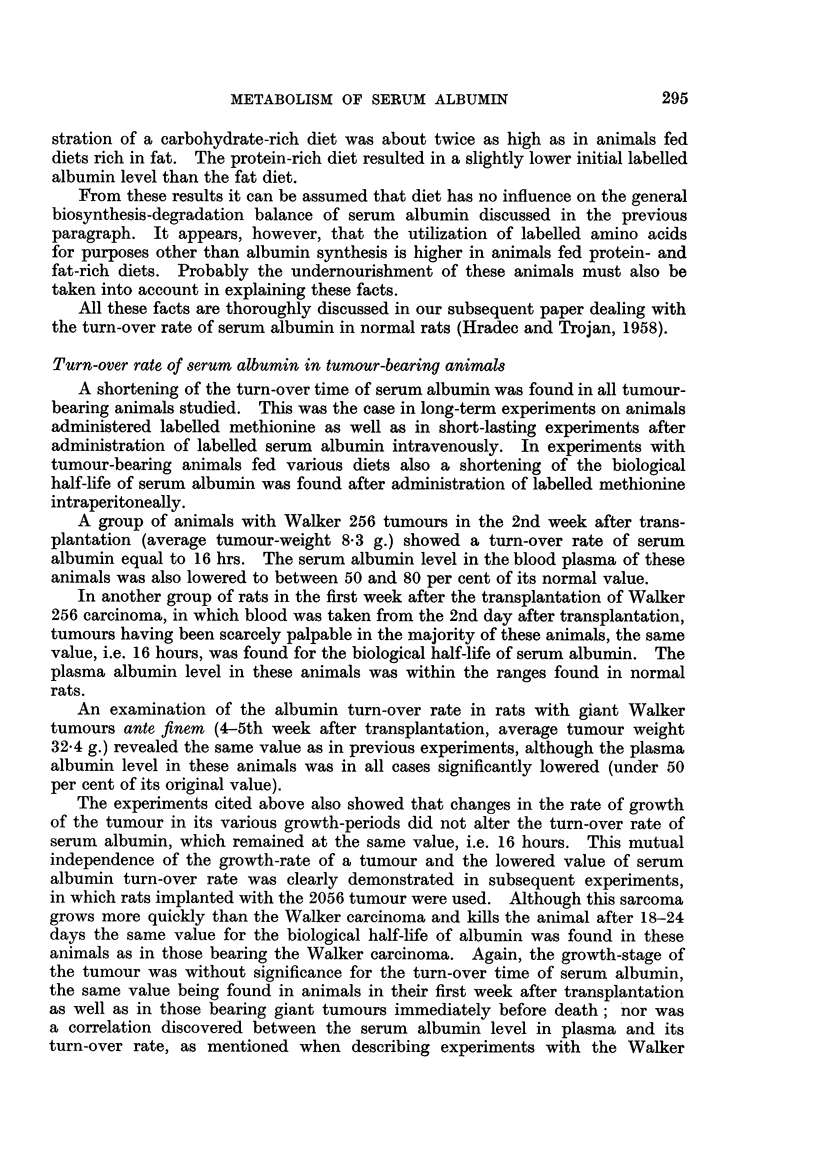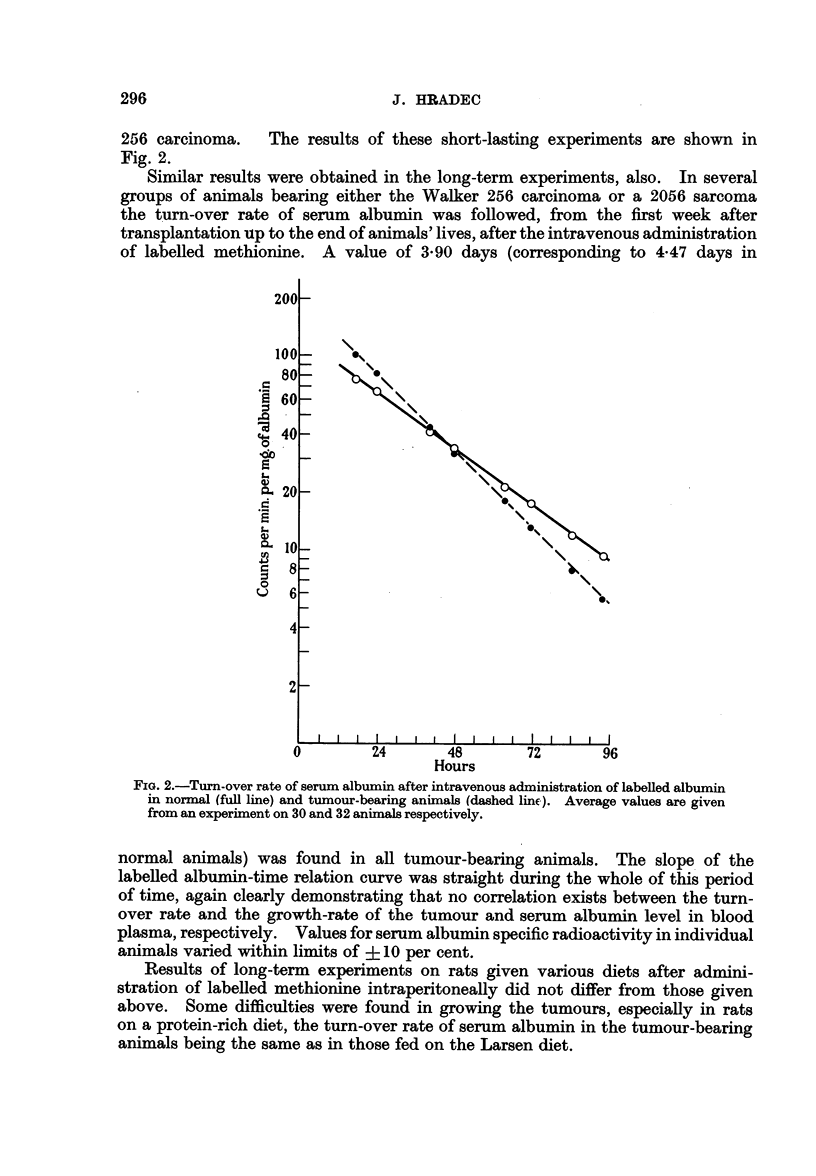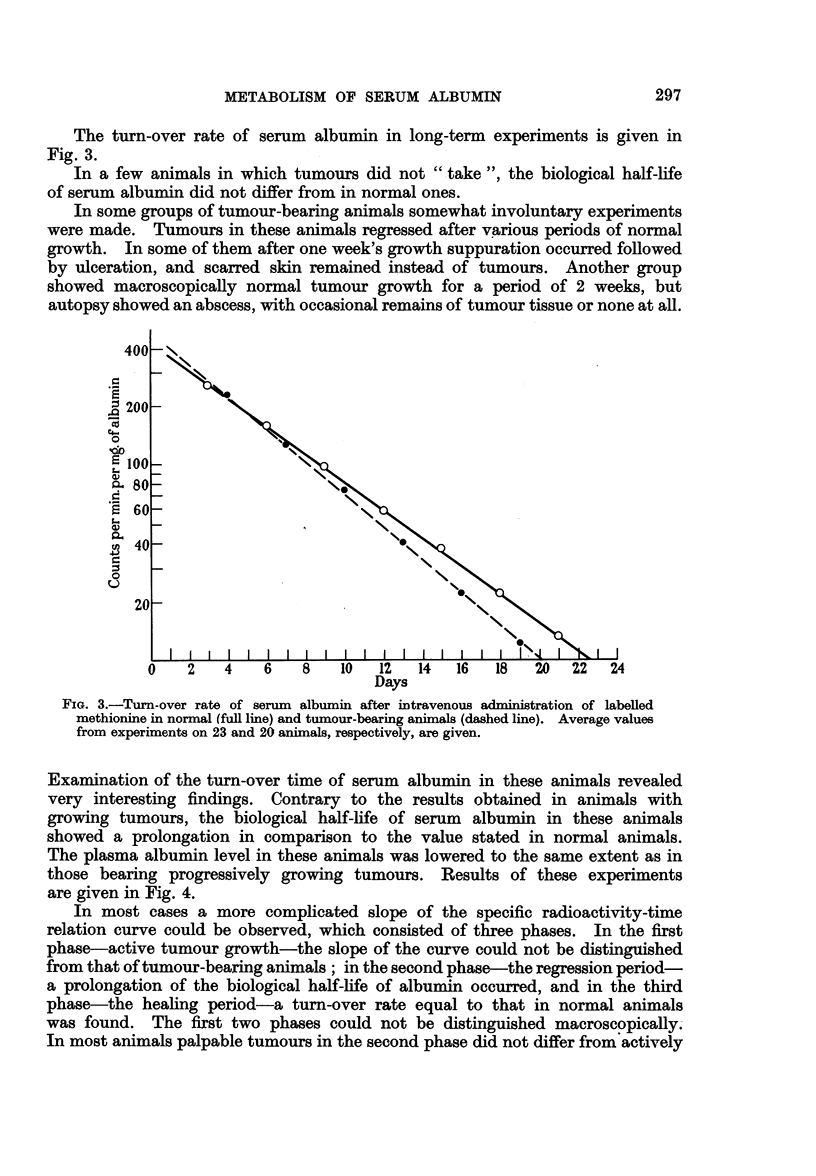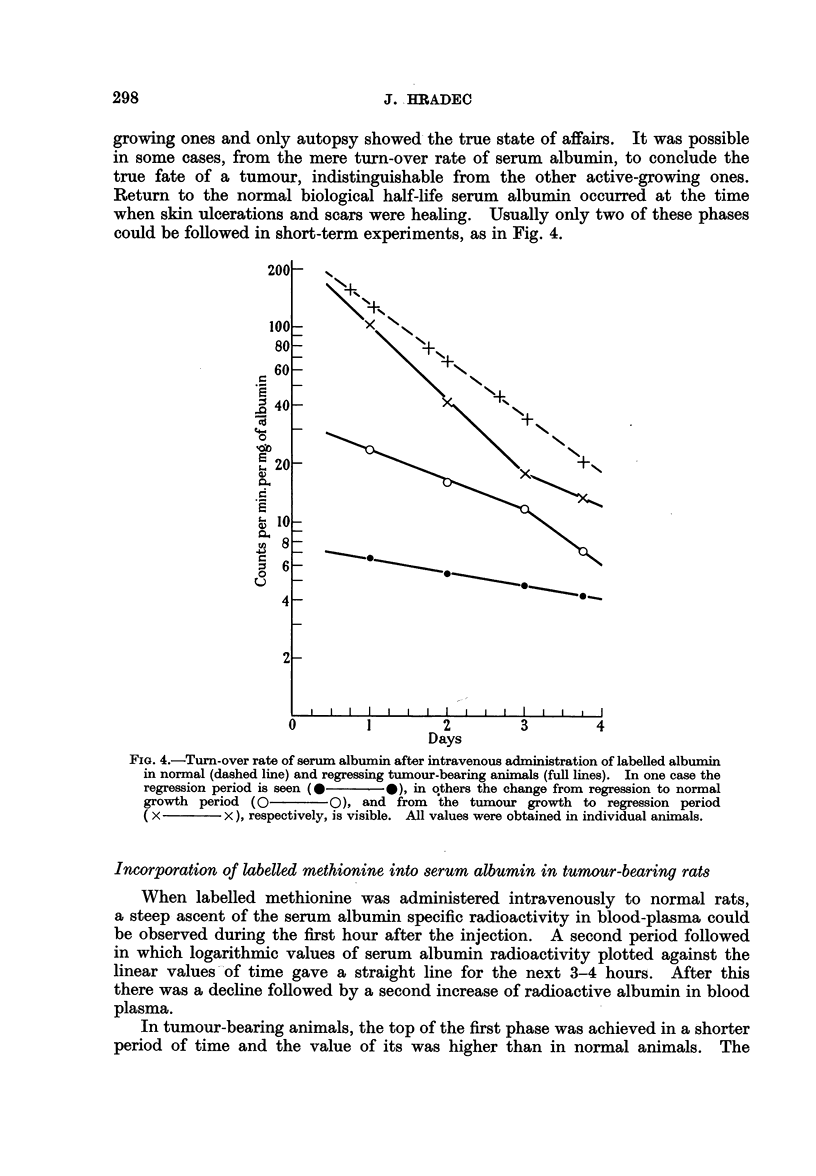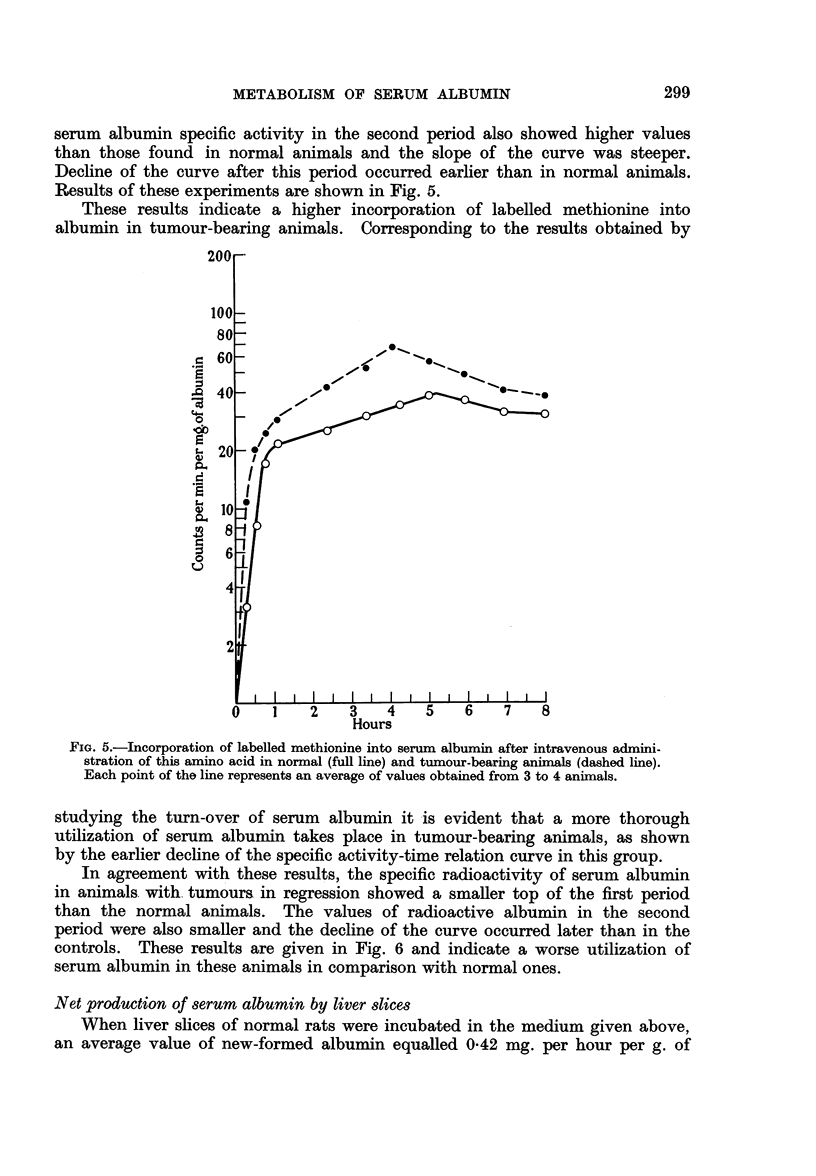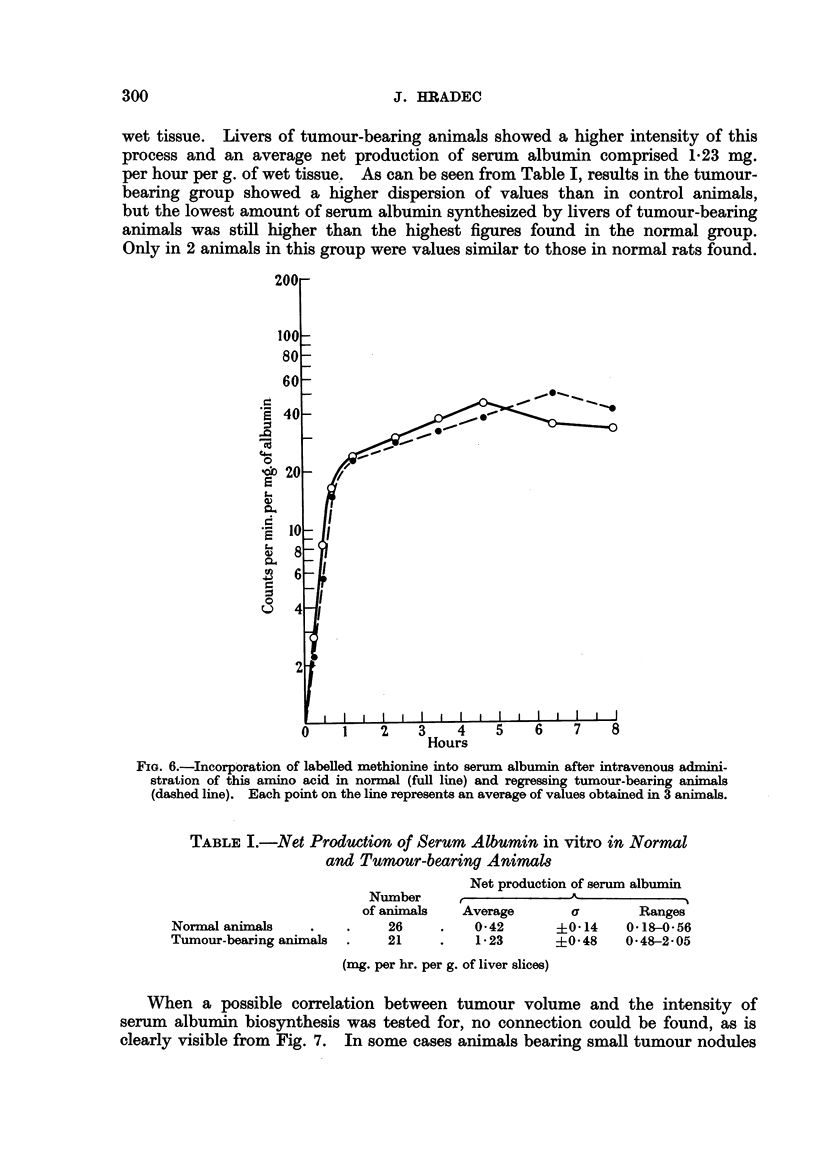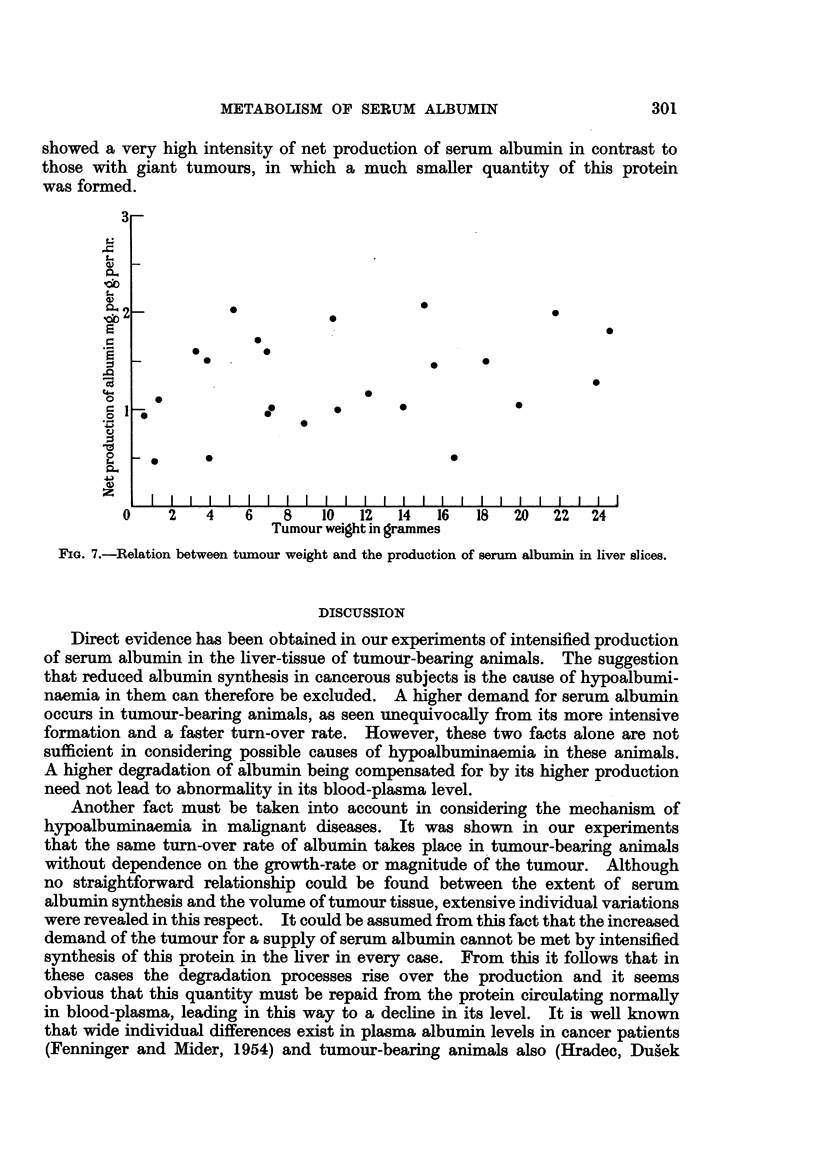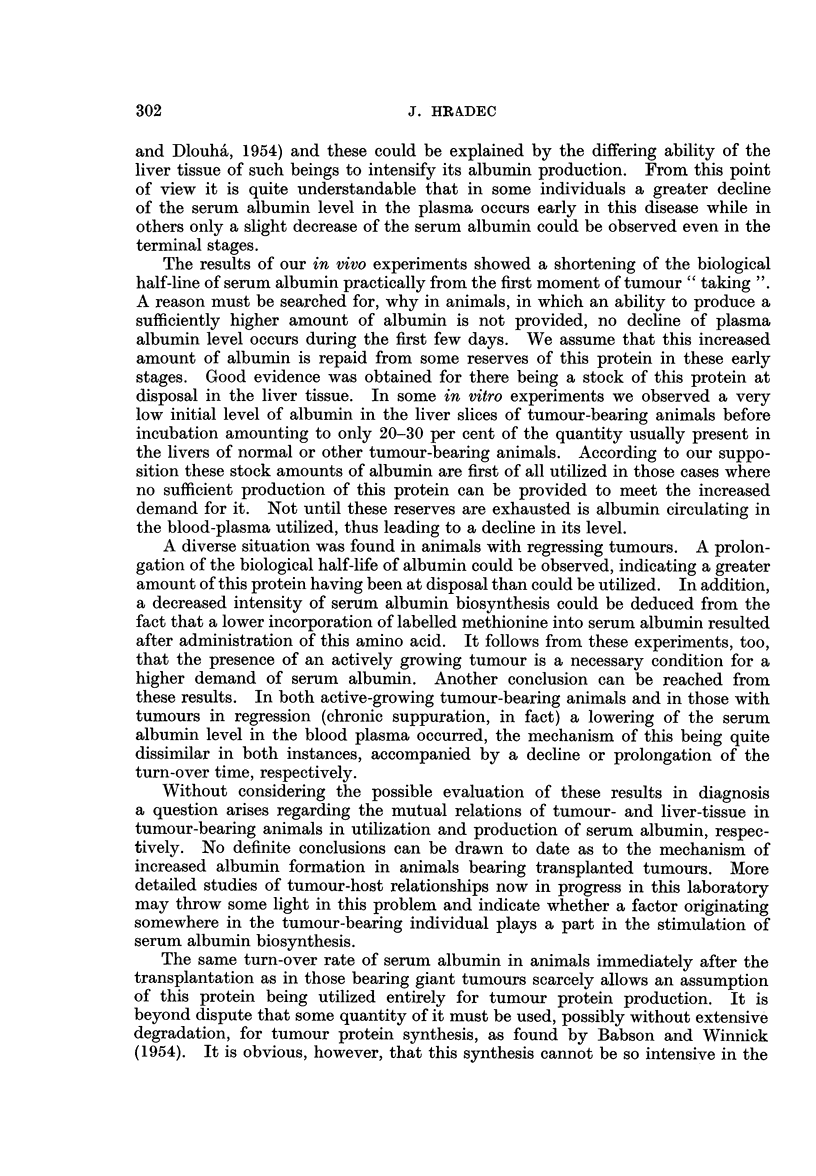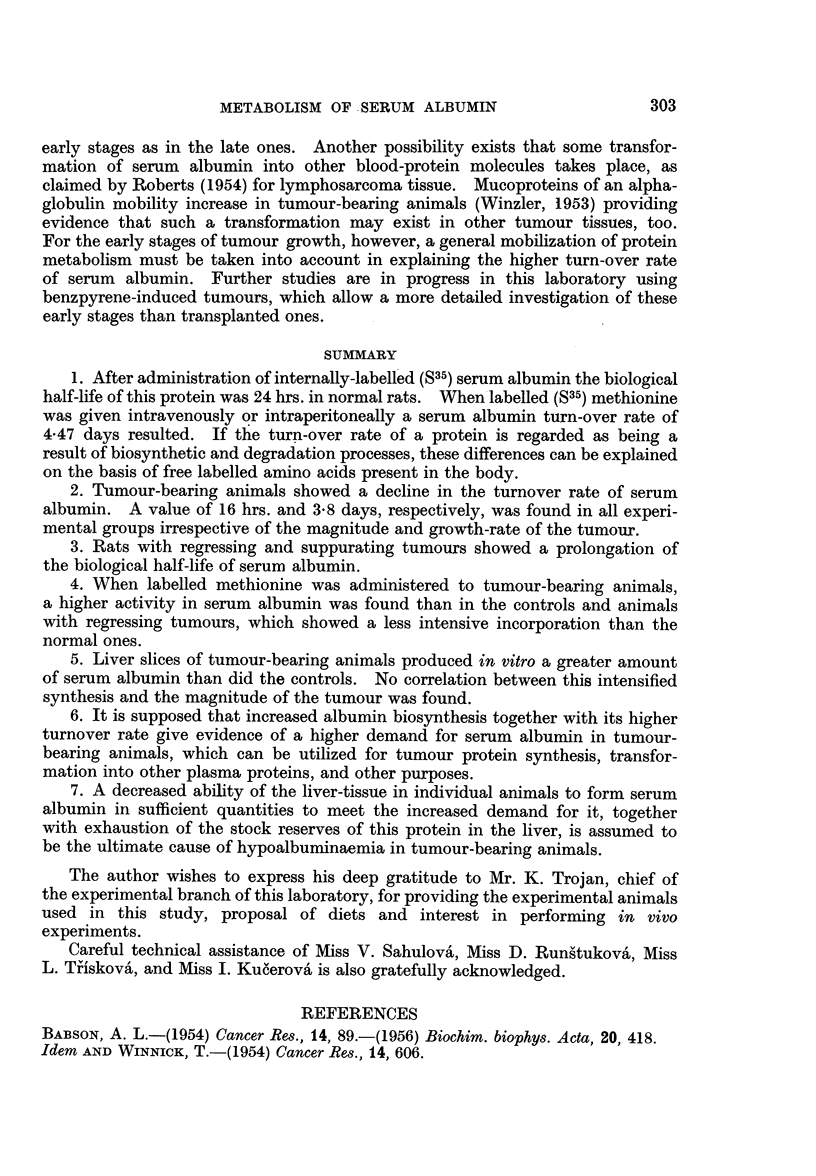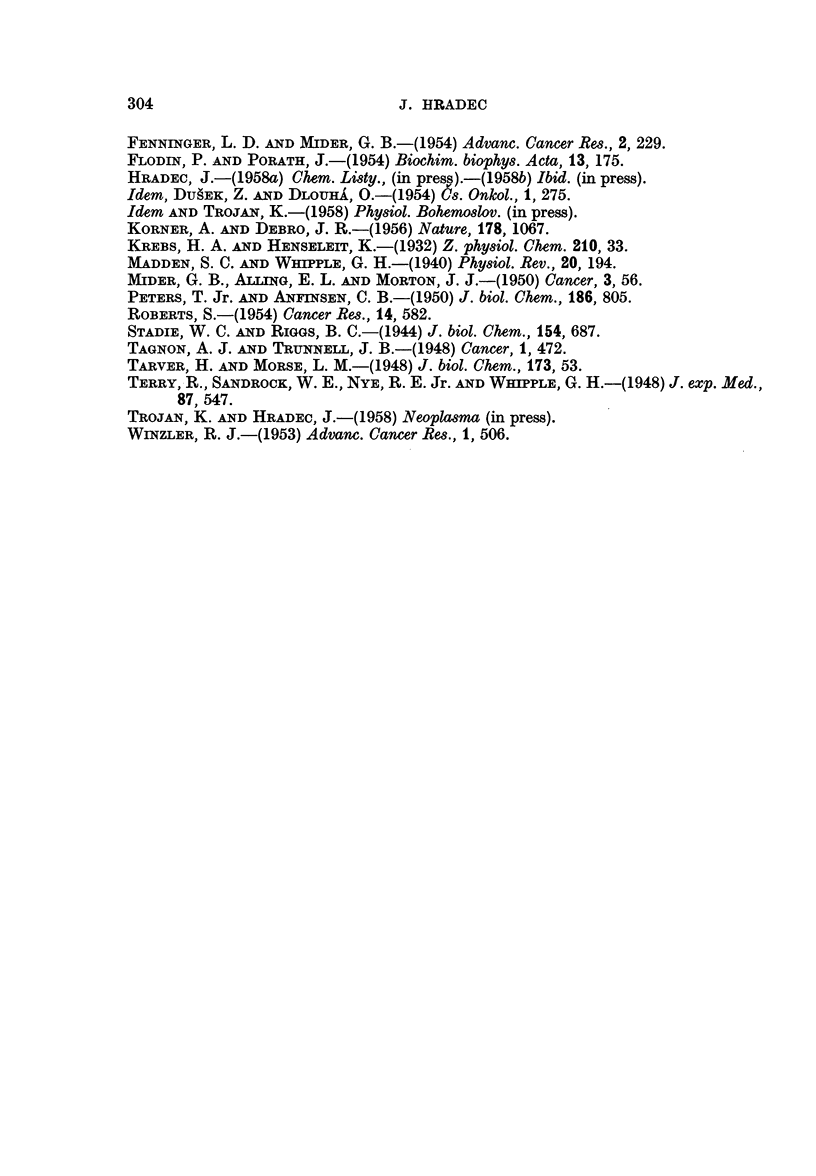# Metabolism of Serum Albumin in Tumour-Bearing Rats

**DOI:** 10.1038/bjc.1958.35

**Published:** 1958-06

**Authors:** J. Hradec


### METABOLISM OF SERUM ALBUMIN IN TUMOUR-BEARING RATS

J. HRADEC

*From the Department of Biochemistry, Oncological Institute, Prague, Czechoslovakia*

Received for publication February 20, 1958

A LOWERING of the serum albumin level is a common accompaniment of malignant disease in man (Mider, Alling and Morton, 1950) and of the growth of experimental tumours in animals (Hradec, Dušek and Dlouhá, 1954). The mechanism whereby this hypoalbuminaemia is produced is still obscure.

Two factors must be taken into account when trying to explain this phenomenon, namely, the intensity of biosynthesis of serum albumin in cancerous subjects, and the extent of utilization of this protein within the body (Fenninger and Mider, 1954). The liver is the main, if not the only, seat of albumin production as shown by experiments with hepatectomized animals (Madden and Whipple, 1940). A net synthesis of this protein has also been demonstrated *in vitro* by incubating liver slices in a suitable medium (Peters and Anfinsen, 1950). Although many studies of liver function have been undertaken in cancer patients and some degree of impairment has been shown in a high percentage of them (Tagnon and Trunnell, 1948), there is as yet no answer to the question whether a decline of serum albumin synthesis in livers of cancerous subjects is the real reason for hypoalbuminaemia occurring in blood. Lack of a suitable definition of this hepatic dysfunction makes it difficult to interpret these results (Fenninger and Mider, 1954). Reports from experiments undertaken in tumour-bearing animals show an enlargement of livers and kidneys while other organs undergo a reduction in their size (Babson, 1954) An impairment of hepatic function can scarcely be supposed in these cases. As it can be seen from these facts, no unequivocal evidence exists up to date that hypoalbuminaemia in malignant diseases is due to the lowered ability of the liver tissue to produce albumin.

There is good evidence for a demand for an increased supply of protein in cancerous subjects. Tumours increase in size by the formation of new protein molecules, for which an increased quantity of building materials must be available. Since the liver holds a key position in the nitrogen metabolism of the body, the suggestion has been made that the liver hypertrophy in rats bearing transplantable tumours is in response to the tumour's demand for an increased supply of protein (Babson, 1954). It is a well-known fact that tissues are able to utilize plasma proteins for the formation of their own proteins, as was demonstrated in dogs given plasma proteins parenterally as the only source of nitrogen (Terry, Sandrock, Nye and Whipple, 1948). Good evidence has also been obtained that a tumour can efficiently utilize plasma proteins for tumour protein production (Babson and Winnick, 1954). In keeping with these assumptions of increased utilization of serum albumin in cancerous subjects is the demonstration of a faster rate of loss of labelled albumin from the circulation of tumour-bearing rats (Babson, 1956). Although no definite conclusions concerning intensified serum albumin synthesis in the livers of these animals can be reached unequivocal evidence was obtained of a higher demand for serum proteins in rats bearing transplanted tumours.

No attempts at correlating these data have so far been reported and no direct experimental proof has as yet been given for an altered serum albumin biosynthesis in tumour-bearing animals. A mere study of the turn-over rate of this protein *in vivo* cannot solve the problem, as at least two factors, degradation and synthesis of new protein molecules, are concerned in experiments of this type. In our laboratory, we tried to study both these factors separately and obtained good evidence that increased intensity of serum albumin formation accompanying a faster rate of loss of this protein from the circulation in tumour-bearing animals may have some connection with the hypoalbuminaemia in these conditions.

### MATERIALS AND METHODS

### *Experimental animals*

A total of 195 Wistar rats of both sexes, weighing 150–200 g., was used throughout our experiments. The animals were housed in glass cages and given tap water *ad libitum.* In most instances, they were fed the semisynthetic Larsen diet (Jelinek, 1952, personal communication), in some cases diets rich in proteins, carbohydrates or fat were given. The protein-rich diet consisted of casein and normal Larsen diet (4 : 1), the carbohydrate diet of starch and normal Larsen diet (4 : 1) and the fat-rich diet contained vegetable fat and normal Larsen diet (1 : 4). Each animal obtained 150 g. of this food daily.

Blood was withdrawn by cardiac puncture under ether anaesthesia, 1·5 ml. being usually taken and mixed with 0·5 ml. of isotonic sodium citrate solution. Bleedings were made on alternate days in short-lasting experiments (usually 3 times from one animal), and twice a week in long term series (6–8 times in one animal). An equivalent volume of saline was given intraperitoneally to all animals bled.

### *Tumours*

The Walker 256 carcinoma and the 2056 sarcoma of this laboratory (Trojan and Hradec, 1958) were used in our experiments. Tumours were implanted by the trocar technique. Growth rates of the tumours were calculated from the volumes of plaster casts prepared on alternate days. The magnitute of the tumour was expressed as the weight of the fresh tumour tissue excised *in toto* from sacrificed animals.

### *Radioactive substances*

Labelled ($S^{35}$) dl-methionine with an average activity of 0·6–0·7 mc. per mg. was obtained from the Radiological Research Institute in Prague. Internally labelled ($S^{35}$) rat serum albumin was prepared biosynthetically in this laboratory and had an activity of 0·3–0·7 uc. per mg. in various batches. It was separated by a cellulose column electrophoresis essentially according to Flodin and Porath (1954) from blood-plasma of rats given intravenously labelled methionine or yeast-protein hydrolysate. Details of this procedure are given in our subsequent paper (Hradec, 1958*a*).

Labelled compounds were administered to animals in most cases intravenously in 0·1–0·2 ml. of isotonic saline solutions. In this case 3–5 mg. of serum albumin or 0·005–0·01 mg. of methionine were given per rat. In some cases labelled methionine or labelled yeast were given orally in diluted milk in doses 0·02–0·05 mg. and 15–20 mg., respectively, per animal.

### In vitro *procedures*

Normal and tumour-bearing animals were killed by a blow on the head, and liver tissue was immediately removed and placed in cold isotonic saline. Tissue slices were made by the Stadie-Riggs slicer (Stadie and Riggs, 1944) and were then suspended in ice-cold saline and shaken three times for two minutes in a mechanical shaking-device, with three or four subsequent washings in ice-cold saline till the washing-fluid was clear and uncoloured (Peters and Anfinsen, 1950). This treatment lowered the blank albumin contents of the tissue to 10–20 per cent of its original value. After this, 0·5 g. of wet slices were suspended in 3 ml. of incubation mixture (Krebs-Ringer bicarbonate (Krebs and Henseleit,1932) without phosphate) in individual Warburg flasks and shaken gently in a Warburg apparatus for 2 hours at 37·5° C. in a gas atmosphere of 95 per cent $O_2$ and 5 per cent $CO_2$. After incubation the contents of each flask were homogenized in a glass homogenizer and the homogenate centrifuged for 10 minutes at 18,000 G. The clear, slightly opalescent supernatant was used for determination of albumin by an isotopic-dilution procedure given next.

### *Chemical methods*

Determination of the serum albumin level in rat plasma was performed by filter paper electrophoresis. A wet-chamber technique, using Barbital-citrate buffer, pH 8·5, $\mu = 0{\cdot}067$, Whatman No. 1 paper and a potential gradient of 8 V per cm. was used. After drying the paper, proteins were stained by bromphenol blue, strips of paper corresponding to each one plasma protein fraction eluted and the percentage of the dye adsorbed determined in a Lange photoelectric colorimeter.

For the determination of the radioactivity of the serum albumin, the plasma proteins were precipitated by trichloracetic acid, the precipitate washed by the samed acid, the albumin extracted with ethanol (Korner and Debro, 1956) and precipitated from this solution by ether. After several washings the precipitate was collected on filter-paper discs and its radioactivity counted. Details of the procedure of albumin isolation are given in our subsequent paper (Hradec, 1958*a*).

Net production of serum albumin in liver slices was determined by an isotope-dilution procedure. Pure labelled albumin was added to supernatants of incubation-mixture homogenates as described above, the proteins were precipitated with trichloracetic acid and serum albumin extracted from the precipitate in the same manner as described in the above paragraph. From the value of specific radioactivity of the washed albumin precipitate the concentration of serum albumin in the sample was calculated using a calibration chart made from specific activities of samples to which known quantities of pure serum albumin had been added. Details of this procedure are given in a subsequent paper (Hradec, 1958*b*). Blank samples for the determination of initial serum albumin level in slices before incubation were prepared in the same way from the washed liver slices as samples incubated. They were prepared immediately before incubation and placed after homogenization and centrifugation in a deep-freezing unit to minimize possible proteolytic processes. Further elaboration of these blank samples then proceeded together with samples after incubation. Values of initial serum albumin level in slices varied between 1·8–3·2 mg. per g. of fresh washed liver slices in different animals, respectively. Usually 4 blank and 6 incubated samples were analysed from the liver of one rat. The statistical error of the determination did not exceed $\pm 2$ per cent in most instances and only cases with a probable error lower than 3 per cent of the blank value were taken into account in our experiments. For the calculation of net serum albumin biosynthesis the blank value was subtracted from the value of incubated samples and results expressed as mg. of serum albumin produced per g. of fresh liver slices per hour.

*Radioactivity assay*

In all cases radioactivity was determined in precipitates finely homogenized in a glass homogenizer and plated on tared filter-paper discs in a filtration device. These samples were counted using a mica-window Geiger-Müller counter from a distance of 20 mm. The counting period chosen was sufficiently long to minimize the probable error to $\pm 2$ per cent. All samples were corrected for decay and self-absorption when necessary.

### RESULTS

*Turn-over rate of serum albumin in normal animals*

Two different values for the biological half-life of serum albumin were obtained depending on the mode of administration of radioactive substances. When labelled methionine was given either orally or by intravenous or intraperitoneal injection, decline of the labelled serum albumin in the blood-plasma of normal animals followed a straight exponential slope from the 7th hour till the 4th week after administration. A more detailed survey of this dependence revealed it to be more complicated with several risings and fallings of the curve. An average value of these results gave 4·47 days for the biological half-life of serum albumin, which is in good agreement with figures given by other authors (Tarver and Morse (1948) 4·4 days).

After administration of labelled pure serum albumin intravenously or labelled yeast protein in food there was a straight exponential decline of the serum albumin specific activity in blood-plasma steeper than in the previous case. This straight line was followed for 3–5 days depending on the quantity of labelled substance administered, after which a temporary rise in the level of labelled serum albumin appeared lasting only a few hours and followed by another decline of the curve. The more radioactive substance was administered the later this ascent of the specific activity-time relation line appeared. The decline of the curve after this rise was more gradual than before. Further temporary ascent of the specific activity appeared after varying periods of time again depending on the quantity of substance administered and after 3 or 4 of these risings the slope of the line was the same as after labelled methionine administration and no additional risings of the curve were found. The value of the biological half-life of serum albumin calculated from the progress of the specific activity-time relation straight line during the first 3 days after the administration of labelled substances gave 24 hours in normal animals.

Biological half-life of serum albumin after various modes of administration of labelled substances is given in Fig. 1.

We assume that no fundamental difference exists between these two seemingly quite dissimilar values for the turn-over time of serum albumin. The specific activity-time relation curve is a result of two processes, the utilization of the protein in the tissues and biosynthesis of new labelled albumin molecules in the body. Where no free labelled amino acids are available for this biosynthesis, the biological half-life is a result of a mere degradation of the labelled protein as in the case of pure albumin administration. In the course of this degradation of

FIG. 1.—The biological half-life of serum albumin in normal rats after intravenous administration of radioactive albumin (full line) or labelled methionine (dashed line). Average values are given, based on the results of an experiment performed on 30 and 23 animals, respectively.

labelled serum albumin, radioactive cystine and methionine are released, which can be utilized for the formation of new serum albumin molecules synthesized in the body. As soon as this occurs, these newly formed protein molecules must be taken into account, in the interpretation of the biological half-life curve. In this way the temporary ascents of the curve when most of the administered albumin has been degraded can be explained, and also the constantly more gradual slope of the labelled albumin level curve. A dynamic balance must be achieved between the processes of albumin degradation and formation, respectively. When this is done, the slope of the curve after about two weeks is exactly the same as in the case of labelled methionine administration, where this balance can be achieved during the first few hours after the administration.

No differences in the turn-over time of serum albumin were found in animals fed on various diets. The slope of the labelled albumin-time relation curve was the same in these animals as in those fed the standard Larsen diet. Significant differences, however, were found in the initial labelled albumin level after feeding labelled methionine. The specific radioactivity of serum albumin after administration of a carbohydrate-rich diet was about twice as high as in animals fed diets rich in fat. The protein-rich diet resulted in a slightly lower initial labelled albumin level than the fat diet.

From these results it can be assumed that diet has no influence on the general biosynthesis-degradation balance of serum albumin discussed in the previous paragraph. It appears, however, that the utilization of labelled amino acids for purposes other than albumin synthesis is higher in animals fed protein- and fat-rich diets. Probably the undernourishment of these animals must also be taken into account in explaining these facts.

All these facts are thoroughly discussed in our subsequent paper dealing with the turn-over rate of serum albumin in normal rats (Hradec and Trojan, 1958).

*Turn-over rate of serum albumin in tumour-bearing animals*

A shortening of the turn-over time of serum albumin was found in all tumour-bearing animals studied. This was the case in long-term experiments on animals administered labelled methionine as well as in short-lasting experiments after administration of labelled serum albumin intravenously. In experiments with tumour-bearing animals fed various diets also a shortening of the biological half-life of serum albumin was found after administration of labelled methionine intraperitoneally.

A group of animals with Walker 256 tumours in the 2nd week after transplantation (average tumour-weight 8·3 g.) showed a turn-over rate of serum albumin equal to 16 hrs. The serum albumin level in the blood plasma of these animals was also lowered to between 50 and 80 per cent of its normal value.

In another group of rats in the first week after the transplantation of Walker 256 carcinoma, in which blood was taken from the 2nd day after transplantation, tumours having been scarcely palpable in the majority of these animals, the same value, i.e. 16 hours, was found for the biological half-life of serum albumin. The plasma albumin level in these animals was within the ranges found in normal rats.

An examination of the albumin turn-over rate in rats with giant Walker tumours *ante finem* (4–5th week after transplantation, average tumour weight 32·4 g.) revealed the same value as in previous experiments, although the plasma albumin level in these animals was in all cases significantly lowered (under 50 per cent of its original value).

The experiments cited above also showed that changes in the rate of growth of the tumour in its various growth-periods did not alter the turn-over rate of serum albumin, which remained at the same value, i.e. 16 hours. This mutual independence of the growth-rate of a tumour and the lowered value of serum albumin turn-over rate was clearly demonstrated in subsequent experiments, in which rats implanted with the 2056 tumour were used. Although this sarcoma grows more quickly than the Walker carcinoma and kills the animal after 18–24 days the same value for the biological half-life of albumin was found in these animals as in those bearing the Walker carcinoma. Again, the growth-stage of the tumour was without significance for the turn-over time of serum albumin, the same value being found in animals in their first week after transplantation as well as in those bearing giant tumours immediately before death ; nor was a correlation discovered between the serum albumin level in plasma and its turn-over rate, as mentioned when describing experiments with the Walker

256 carcinoma. The results of these short-lasting experiments are shown in Fig. 2.

Similar results were obtained in the long-term experiments, also. In several groups of animals bearing either the Walker 256 carcinoma or a 2056 sarcoma the turn-over rate of serum albumin was followed, from the first week after transplantation up to the end of animals' lives, after the intravenous administration of labelled methionine. A value of 3·90 days (corresponding to 4·47 days in

Fig. 2.—Turn-over rate of serum albumin after intravenous administration of labelled albumin in normal (full line) and tumour-bearing animals (dashed line). Average values are given from an experiment on 30 and 32 animals respectively.

normal animals) was found in all tumour-bearing animals. The slope of the labelled albumin-time relation curve was straight during the whole of this period of time, again clearly demonstrating that no correlation exists between the turn-over rate and the growth-rate of the tumour and serum albumin level in blood plasma, respectively. Values for serum albumin specific radioactivity in individual animals varied within limits of $\pm 10$ per cent.

Results of long-term experiments on rats given various diets after administration of labelled methionine intraperitoneally did not differ from those given above. Some difficulties were found in growing the tumours, especially in rats on a protein-rich diet, the turn-over rate of serum albumin in the tumour-bearing animals being the same as in those fed on the Larsen diet.

The turn-over rate of serum albumin in long-term experiments is given in Fig. 3.

In a few animals in which tumours did not " take ", the biological half-life of serum albumin did not differ from in normal ones.

In some groups of tumour-bearing animals somewhat involuntary experiments were made. Tumours in these animals regressed after various periods of normal growth. In some of them after one week's growth suppuration occurred followed by ulceration, and scarred skin remained instead of tumours. Another group showed macroscopically normal tumour growth for a period of 2 weeks, but autopsy showed an abscess, with occasional remains of tumour tissue or none at all.

FIG. 3.—Turn-over rate of serum albumin after intravenous administration of labelled methionine in normal (full line) and tumour-bearing animals (dashed line). Average values from experiments on 23 and 20 animals, respectively, are given.

Examination of the turn-over time of serum albumin in these animals revealed very interesting findings. Contrary to the results obtained in animals with growing tumours, the biological half-life of serum albumin in these animals showed a prolongation in comparison to the value stated in normal animals. The plasma albumin level in these animals was lowered to the same extent as in those bearing progressively growing tumours. Results of these experiments are given in Fig. 4.

In most cases a more complicated slope of the specific radioactivity-time relation curve could be observed, which consisted of three phases. In the first phase—active tumour growth—the slope of the curve could not be distinguished from that of tumour-bearing animals ; in the second phase—the regression period—a prolongation of the biological half-life of albumin occurred, and in the third phase—the healing period—a turn-over rate equal to that in normal animals was found. The first two phases could not be distinguished macroscopically. In most animals palpable tumours in the second phase did not differ from actively growing ones and only autopsy showed the true state of affairs. It was possible in some cases, from the mere turn-over rate of serum albumin, to conclude the true fate of a tumour, indistinguishable from the other active-growing ones. Return to the normal biological half-life serum albumin occurred at the time when skin ulcerations and scars were healing. Usually only two of these phases could be followed in short-term experiments, as in Fig. 4.

FIG. 4.—Turn-over rate of serum albumin after intravenous administration of labelled albumin in normal (dashed line) and regressing tumour-bearing animals (full lines). In one case the regression period is seen (●———●), in others the change from regression to normal growth period (○———○), and from the tumour growth to regression period (×———×), respectively, is visible. All values were obtained in individual animals.

*Incorporation of labelled methionine into serum albumin in tumour-bearing rats*

When labelled methionine was administered intravenously to normal rats, a steep ascent of the serum albumin specific radioactivity in blood-plasma could be observed during the first hour after the injection. A second period followed in which logarithmic values of serum albumin radioactivity plotted against the linear values of time gave a straight line for the next 3–4 hours. After this there was a decline followed by a second increase of radioactive albumin in blood plasma.

In tumour-bearing animals, the top of the first phase was achieved in a shorter period of time and the value of its was higher than in normal animals. The serum albumin specific activity in the second period also showed higher values than those found in normal animals and the slope of the curve was steeper. Decline of the curve after this period occurred earlier than in normal animals. Results of these experiments are shown in Fig. 5.

These results indicate a higher incorporation of labelled methionine into albumin in tumour-bearing animals. Corresponding to the results obtained by

FIG. 5.—Incorporation of labelled methionine into serum albumin after intravenous administration of this amino acid in normal (full line) and tumour-bearing animals (dashed line). Each point of the line represents an average of values obtained from 3 to 4 animals.

studying the turn-over of serum albumin it is evident that a more thorough utilization of serum albumin takes place in tumour-bearing animals, as shown by the earlier decline of the specific activity-time relation curve in this group.

In agreement with these results, the specific radioactivity of serum albumin in animals with tumours in regression showed a smaller top of the first period than the normal animals. The values of radioactive albumin in the second period were also smaller and the decline of the curve occurred later than in the controls. These results are given in Fig. 6 and indicate a worse utilization of serum albumin in these animals in comparison with normal ones.

*Net production of serum albumin by liver slices*

When liver slices of normal rats were incubated in the medium given above, an average value of new-formed albumin equalled 0·42 mg. per hour per g. of wet tissue. Livers of tumour-bearing animals showed a higher intensity of this process and an average net production of serum albumin comprised 1·23 mg. per hour per g. of wet tissue. As can be seen from Table I, results in the tumour-bearing group showed a higher dispersion of values than in control animals, but the lowest amount of serum albumin synthesized by livers of tumour-bearing animals was still higher than the highest figures found in the normal group. Only in 2 animals in this group were values similar to those in normal rats found.

FIG. 6.—Incorporation of labelled methionine into serum albumin after intravenous administration of this amino acid in normal (full line) and regressing tumour-bearing animals (dashed line). Each point on the line represents an average of values obtained in 3 animals.

TABLE I.—*Net Production of Serum Albumin* in vitro *in Normal and Tumour-bearing Animals*

| | Number of animals | Net production of serum albumin | | |
|---|---|---|---|---|
| | | Average | $\sigma$ | Ranges |
| Normal animals | 26 | 0·42 | ±0·14 | 0·18–0·56 |
| Tumour-bearing animals | 21 | 1·23 | ±0·48 | 0·48–2·05 |

(mg. per hr. per g. of liver slices)

When a possible correlation between tumour volume and the intensity of serum albumin biosynthesis was tested for, no connection could be found, as is clearly visible from Fig. 7. In some cases animals bearing small tumour nodules showed a very high intensity of net production of serum albumin in contrast to those with giant tumours, in which a much smaller quantity of this protein was formed.

FIG. 7.—Relation between tumour weight and the production of serum albumin in liver slices.

### DISCUSSION

Direct evidence has been obtained in our experiments of intensified production of serum albumin in the liver-tissue of tumour-bearing animals. The suggestion that reduced albumin synthesis in cancerous subjects is the cause of hypoalbuminaemia in them can therefore be excluded. A higher demand for serum albumin occurs in tumour-bearing animals, as seen unequivocally from its more intensive formation and a faster turn-over rate. However, these two facts alone are not sufficient in considering possible causes of hypoalbuminaemia in these animals. A higher degradation of albumin being compensated for by its higher production need not lead to abnormality in its blood-plasma level.

Another fact must be taken into account in considering the mechanism of hypoalbuminaemia in malignant diseases. It was shown in our experiments that the same turn-over rate of albumin takes place in tumour-bearing animals without dependence on the growth-rate or magnitude of the tumour. Although no straightforward relationship could be found between the extent of serum albumin synthesis and the volume of tumour tissue, extensive individual variations were revealed in this respect. It could be assumed from this fact that the increased demand of the tumour for a supply of serum albumin cannot be met by intensified synthesis of this protein in the liver in every case. From this it follows that in these cases the degradation processes rise over the production and it seems obvious that this quantity must be repaid from the protein circulating normally in blood-plasma, leading in this way to a decline in its level. It is well known that wide individual differences exist in plasma albumin levels in cancer patients (Fenninger and Mider, 1954) and tumour-bearing animals also (Hradec, Dušek and Dlouhá, 1954) and these could be explained by the differing ability of the liver tissue of such beings to intensify its albumin production. From this point of view it is quite understandable that in some individuals a greater decline of the serum albumin level in the plasma occurs early in this disease while in others only a slight decrease of the serum albumin could be observed even in the terminal stages.

The results of our *in vivo* experiments showed a shortening of the biological half-line of serum albumin practically from the first moment of tumour " taking ". A reason must be searched for, why in animals, in which an ability to produce a sufficiently higher amount of albumin is not provided, no decline of plasma albumin level occurs during the first few days. We assume that this increased amount of albumin is repaid from some reserves of this protein in these early stages. Good evidence was obtained for there being a stock of this protein at disposal in the liver tissue. In some *in vitro* experiments we observed a very low initial level of albumin in the liver slices of tumour-bearing animals before incubation amounting to only 20–30 per cent of the quantity usually present in the livers of normal or other tumour-bearing animals. According to our supposition these stock amounts of albumin are first of all utilized in those cases where no sufficient production of this protein can be provided to meet the increased demand for it. Not until these reserves are exhausted is albumin circulating in the blood-plasma utilized, thus leading to a decline in its level.

A diverse situation was found in animals with regressing tumours. A prolongation of the biological half-life of albumin could be observed, indicating a greater amount of this protein having been at disposal than could be utilized. In addition, a decreased intensity of serum albumin biosynthesis could be deduced from the fact that a lower incorporation of labelled methionine into serum albumin resulted after administration of this amino acid. It follows from these experiments, too, that the presence of an actively growing tumour is a necessary condition for a higher demand of serum albumin. Another conclusion can be reached from these results. In both active-growing tumour-bearing animals and in those with tumours in regression (chronic suppuration, in fact) a lowering of the serum albumin level in the blood plasma occurred, the mechanism of this being quite dissimilar in both instances, accompanied by a decline or prolongation of the turn-over time, respectively.

Without considering the possible evaluation of these results in diagnosis a question arises regarding the mutual relations of tumour- and liver-tissue in tumour-bearing animals in utilization and production of serum albumin, respectively. No definite conclusions can be drawn to date as to the mechanism of increased albumin formation in animals bearing transplanted tumours. More detailed studies of tumour-host relationships now in progress in this laboratory may throw some light in this problem and indicate whether a factor originating somewhere in the tumour-bearing individual plays a part in the stimulation of serum albumin biosynthesis.

The same turn-over rate of serum albumin in animals immediately after the transplantation as in those bearing giant tumours scarcely allows an assumption of this protein being utilized entirely for tumour protein production. It is beyond dispute that some quantity of it must be used, possibly without extensive degradation, for tumour protein synthesis, as found by Babson and Winnick (1954). It is obvious, however, that this synthesis cannot be so intensive in the early stages as in the late ones. Another possibility exists that some transformation of serum albumin into other blood-protein molecules takes place, as claimed by Roberts (1954) for lymphosarcoma tissue. Mucoproteins of an alpha-globulin mobility increase in tumour-bearing animals (Winzler, 1953) providing evidence that such a transformation may exist in other tumour tissues, too. For the early stages of tumour growth, however, a general mobilization of protein metabolism must be taken into account in explaining the higher turn-over rate of serum albumin. Further studies are in progress in this laboratory using benzpyrene-induced tumours, which allow a more detailed investigation of these early stages than transplanted ones.

### SUMMARY

1. After administration of internally-labelled ($S^{35}$) serum albumin the biological half-life of this protein was 24 hrs. in normal rats. When labelled ($S^{35}$) methionine was given intravenously or intraperitoneally a serum albumin turn-over rate of 4·47 days resulted. If the turn-over rate of a protein is regarded as being a result of biosynthetic and degradation processes, these differences can be explained on the basis of free labelled amino acids present in the body.

2. Tumour-bearing animals showed a decline in the turnover rate of serum albumin. A value of 16 hrs. and 3·8 days, respectively, was found in all experimental groups irrespective of the magnitude and growth-rate of the tumour.

3. Rats with regressing and suppurating tumours showed a prolongation of the biological half-life of serum albumin.

4. When labelled methionine was administered to tumour-bearing animals, a higher activity in serum albumin was found than in the controls and animals with regressing tumours, which showed a less intensive incorporation than the normal ones.

5. Liver slices of tumour-bearing animals produced *in vitro* a greater amount of serum albumin than did the controls. No correlation between this intensified synthesis and the magnitude of the tumour was found.

6. It is supposed that increased albumin biosynthesis together with its higher turnover rate give evidence of a higher demand for serum albumin in tumour-bearing animals, which can be utilized for tumour protein synthesis, transformation into other plasma proteins, and other purposes.

7. A decreased ability of the liver-tissue in individual animals to form serum albumin in sufficient quantities to meet the increased demand for it, together with exhaustion of the stock reserves of this protein in the liver, is assumed to be the ultimate cause of hypoalbuminaemia in tumour-bearing animals.

The author wishes to express his deep gratitude to Mr. K. Trojan, chief of the experimental branch of this laboratory, for providing the experimental animals used in this study, proposal of diets and interest in performing *in vivo* experiments.

Careful technical assistance of Miss V. Sahulová, Miss D. Runštuková, Miss L. Třísková, and Miss I. Kučerová is also gratefully acknowledged.

### REFERENCES

BABSON, A. L.—(1954) *Cancer Res.*, **14**, 89.—(1956) *Biochim. biophys. Acta*, **20**, 418.
*Idem* AND WINNICK, T.—(1954) *Cancer Res.*, **14**, 606.

Fenninger, L. D. and Mider, G. B.—(1954) *Advanc. Cancer Res.*, **2**, 229.
Flodin, P. and Porath, J.—(1954) *Biochim. biophys. Acta*, **13**, 175.
Hradec, J.—(1958*a*) *Chem. Listy.*, (in press).—(1958*b*) *Ibid.* (in press).
*Idem*, Dušek, Z. and Dlouhá, O.—(1954) *Čs. Onkol.*, **1**, 275.
*Idem* and Trojan, K.—(1958) *Physiol. Bohemoslov.* (in press).
Korner, A. and Debro, J. R.—(1956) *Nature*, **178**, 1067.
Krebs, H. A. and Henseleit, K.—(1932) *Z. physiol. Chem.* **210**, 33.
Madden, S. C. and Whipple, G. H.—(1940) *Physiol. Rev.*, **20**, 194.
Mider, G. B., Alling, E. L. and Morton, J. J.—(1950) *Cancer*, **3**, 56.
Peters, T. Jr. and Anfinsen, C. B.—(1950) *J. biol. Chem.*, **186**, 805.
Roberts, S.—(1954) *Cancer Res.*, **14**, 582.
Stadie, W. C. and Riggs, B. C.—(1944) *J. biol. Chem.*, **154**, 687.
Tagnon, A. J. and Trunnell, J. B.—(1948) *Cancer*, **1**, 472.
Tarver, H. and Morse, L. M.—(1948) *J. biol. Chem.*, **173**, 53.
Terry, R., Sandrock, W. E., Nye, R. E. Jr. and Whipple, G. H.—(1948) *J. exp. Med.*, **87**, 547.
Trojan, K. and Hradec, J.—(1958) *Neoplasma* (in press).
Winzler, R. J.—(1953) *Advanc. Cancer Res.*, **1**, 506.